# High-Risk Plaque Characteristics in Patients with Suspected Stable Coronary Artery Disease and Impaired Glucose Tolerance: A Coronary Computed Tomography Angiography Study

**DOI:** 10.3390/jcdd12020037

**Published:** 2025-01-22

**Authors:** Thomas Rueskov Andersen, Katrine Schultz Overgaard, Laurits Juhl Heinsen, Roda Abdulkadir Mohamed, Freja Sønder Madsen, Helle Precht, Jess Lambrechtsen, Søren Auscher, Kenneth Egstrup

**Affiliations:** 1 Cardiovascular Research Unit, Odense University Hospital Svendborg, 5700 Svendborg, Denmark; thomas.ruekov.andersen@rsyd.dk (T.R.A.); katrine.schultz.overgaard@rsyd.dk (K.S.O.); laurits.juhl.heinsen@rsyd.dk (L.J.H.); roda.abdulkadir.mohamed@rsyd.dk (R.A.M.); freja.sonder.madsen@rsyd.dk (F.S.M.); hepr@ucl.dk (H.P.); jess.lambrechtsen@rsyd.dk (J.L.); soeren.auscher@rsyd.dk (S.A.); 2 Department of Clinical Research, Faculty of Health Sciences, University of Southern Denmark, 5230 Odense, Denmark; 3 Health Sciences Research Centre, UCL University College, 5230 Odense, Denmark; 4 Department of Radiology, Lillebaelt Hospital, University Hospitals of Southern Denmark, 6000 Kolding, Denmark; 5 Institute of Regional Health Research, University of Southern Denmark, 5230 Odense, Denmark; 6 Discipline of Medical Imaging and Radiation Therapy, University College Cork, T12 K8AF Cork, Ireland

**Keywords:** low-attenuation plaque, high-risk plaque feature, coronary CT angiography, coronary artery disease, impaired glucose tolerance, prediabetes

## Abstract

Impaired glucose tolerance (IGT), a prediabetic state, is a known risk factor for coronary artery disease (CAD). Low-attenuation plaque (LAP) lesions are associated with a high risk of coronary events. We aimed to evaluate high-risk plaque characteristics in LAP lesions between patients with IGT and normal glucose tolerance (NGT) in patients suspected for stable CAD. Coronary computed tomography angiography (CCTA) identified LAP lesions and assessed plaque volumes, burdens, and high-risk plaque features. Glycemic tolerance was stratified using oral glucose tolerance tests. Among 148 patients, 202 LAP lesions were identified, with 93 patients classified as NGT and 55 as IGT. Patients with IGT had a significantly higher prevalence of LAP lesions compared with NGT (*p* = 0.007). LAP volume was higher in IGT (16.46 ± 12.52 mm^3^) compared with NGT (12.66 ± 9.72 mm^3^, *p* = 0.01), but this association did not persist in multivariate analysis. The LAP burden was greater in IGT (10.79 ± 6.84%) than NGT (8.62 ± 5.93%, *p* = 0.02), and the napkin-ring sign was more frequent in IGT (12%) versus NGT (5%, *p* = 0.02); these associations remained significant in multivariate analysis. Patients with IGT had a higher LAP burden and higher frequency of napkin-ring signs. These findings may help explain the common occurrence of prediabetes in patients with acute myocardial infarction.

## 1. Introduction

Impaired glucose tolerance (IGT), an intermediate state between normal glucose metabolism and type 2 diabetes mellitus (T2DM), is a well-established risk factor for coronary artery disease (CAD) [1]. As part of the broader category of prediabetes, which also includes impaired fasting glucose (IFG), IGT has shown a particularly strong association with adverse CAD outcomes [2]. While prediabetes can be assessed using glycated hemoglobinA1c (HbA1c) levels, 2-h plasma glucose (2-hPG) levels ≥ 9 mmol/L are superior for predicting CAD progression [3]. This emphasizes the importance of using the oral glucose tolerance test (OGTT) as a primary screening tool to identify patients at elevated risk [3,4].

High-risk plaque characteristics, which are crucial determinants of cardiovascular (CV) outcomes, can be assessed using coronary computed tomography angiography (CCTA), a simple and non-invasive method. Understanding the relationship between IGT and plaque vulnerability is essential for effective risk stratification [5,6,7]. Low-attenuation plaques (LAPs), typically defined by Hounsfield Units (HUs) less than 30, are indicative of lipid-rich necrotic cores within atherosclerotic plaque [8]. Patients with acute coronary syndrome (ACS) often exhibit LAP lesions, which are associated with an increased risk of future CV events [9,10,11]. Additionally, patients who exhibit high-risk plaque (HRP) features, such as spotty calcification (SC), positive remodeling index (RI), and the napkin-ring sign (NRS), are at an increased risk of adverse CV outcomes [12].

Recent evidence from optical coherence tomography (OCT) studies has demonstrated that coronary plaques in patients with IGT exhibit a greater lipid core size and thinner fibrous caps, suggesting increased vulnerability similar to that observed in patients with T2DM [5,13]. Despite these findings, a significant knowledge gap remains in understanding how IGT contributes to high-risk plaque characteristics measured using CCTA.

In this observational study of patients with coronary low-attenuation plaque lesions, we compared the high-risk plaque characteristics, comprising plaque volumes, burdens, and high-risk plaque features in patients with normal and impaired glucose tolerance.

## 2. Materials and Methods

### 2.1. Study Design

This observational, prospective, single-center study included a blinded comparison between glycemic tolerance and coronary high-risk plaque characteristics. The study was conducted at the Outpatient Clinic of Cardiology and the Department of Cardiovascular Research at Odense University Hospital, Svendborg. We enrolled patients referred for CCTA due to suspected stable CAD between February 2018 and June 2020. All participants provided written informed consent, and the study received approval from the Regional Scientific Ethics Committee for Southern Denmark (project ID: S-20170094) and the Danish Data Protection Agency (project ID: 2012-58-0018).

### 2.2. Study Population

Patients were invited to participate during their CCTA visit and were subsequently scheduled for a consultation, during which written consent was obtained, medical history reviewed, and blood samples collected. The mean interval between CCTA and blood sampling was 29 days. Inclusion criteria were age ≥ 18 years, capacity to provide informed consent, and suspicion of stable CAD. Exclusion criteria included conditions unsuitable for contrast-enhanced CCTA, such as body mass index (BMI) > 40, irregular or fast heart rhythms incompatible with CCTA, reduced kidney function with an estimated glomerular filtration rate (eGFR) < 45 mL/min, and known contrast allergy.

**Figure 1:**
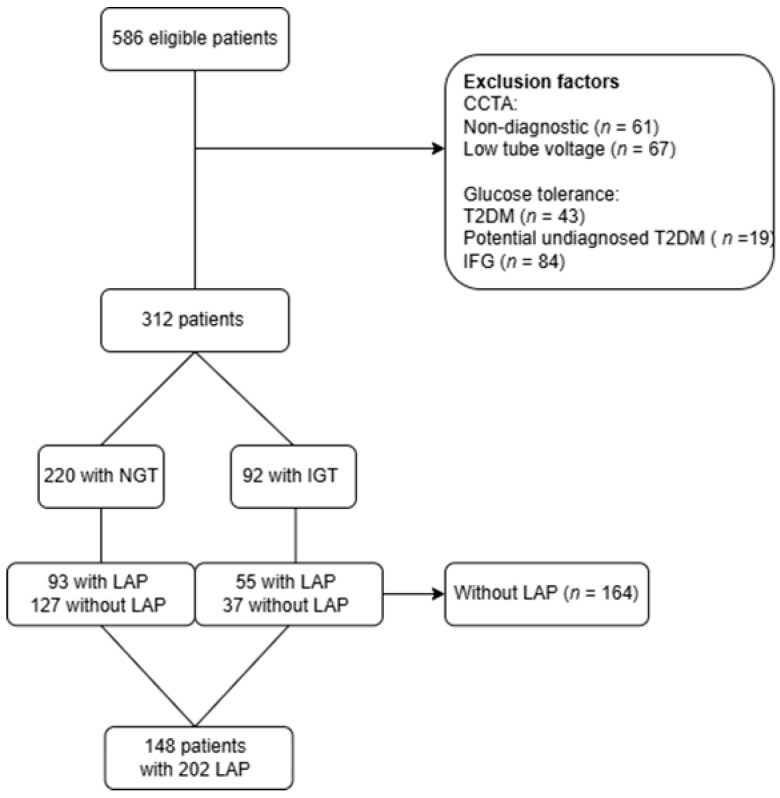
CONSORT flowchart. This flowchart illustrates the process of study sample selection, detailing patient eligibility based on exclusion criteria for CCTA scan analysis, glucose tolerance, and the presence of low-attenuation plaque lesions. CCTA = coronary CT angiography, n = number, T2DM = Type 2 Diabetes Mellitus, IFG = impaired fasting glucose, NGT = normal glucose tolerance, IGT = impaired glucose tolerance, LAP = low-attenuation plaque.

A total of 586 consecutive patients with suspected CAD were initially enrolled (Figure 1). Patients with non-diagnostic CCTA scans were excluded (*n* = 61), and those scanned with low tube voltage were excluded for comparability reasons, as shown by Takagi et al. [14]. Next, glucose tolerance was evaluated and those with a T2DM diagnosis were excluded. For transparency, the characteristics and plaque data of T2DM patients, alongside those of NGT and IGT patients, have been included in the supplemental material (Tables S1 and S2). To ensure the exclusion of potentially undiagnosed T2DM, individuals with fasting plasma glucose (FPG) levels > 7.0 mmol/L or 2-h glucose values ≥ 11.1 mmol/L were excluded. Additionally, patients with IFG were excluded because of their high degree of similarity to those with normal glucose tolerance (NGT). This resulted in 312 patients, including 220 with NGT and 92 with IGT. Figure 1 illustrates the process of the study sample selection. Lesions with LAP were present in 93 of the 220 patients with NGT and 55 of the 92 patients with IGT, while those without a LAP lesion were excluded (*n* = 164).

### 2.3. Clinical and Biochemical Assessments

A questionnaire was used to evaluate CV risk factors, encompassing age, height, weight, smoking status, history of CAD, medical history, and medication use. Blood samples were obtained and assessed for various biomarkers, including HbA1c, FPG, 120 min plasma glucose, total cholesterol, low-density lipoprotein (LDL), high-density lipoprotein (HDL), triglycerides, eGFR, creatinine, and C-reactive protein (CRP). HbA1c was categorized as pre-diabetic using the threshold of 39–47 mmol/mol from the American Diabetes Association (ADA) [15].

### 2.4. Glucose Tolerance Testing and Stratification

OGTT was performed after CCTA in patients without known T2DM. Patients were instructed to fast for 8 h before the OGTT. FPG was measured before 75 g of glucose was ingested over five minutes. After 120 min, plasma glucose was measured, and patients were stratified according to ADA definitions of glycemic status [15].

Patients were stratified into the following groups based on their OGTT results: NGT and IGT.

### 2.5. CCTA Acquisition

CCTA images were obtained using a standardized protocol on a 256-detector system (GE-revolution APEX CT, GE Healthcare, Waukesha, WI, USA). We also conducted an unenhanced scan to assess coronary artery calcium. Ivabradine 7.5 mg tablets were administered, one tablet the night before the scan and one tablet in the morning on scanning day to improve image quality. If necessary, intravenous beta-blockers were administered on the day of the scan. ECG-gated prospective acquisition in the 75% R-R interval, with additional padding of 45 ms to allow for additional construction, was used to obtain images. A total of 60 mL of iodine contrast (Visiplaque 320 mg iodine/mL) was injected at a rate of 5 mL/s, and the scan was timed when maximum attenuation was detected in the descending aorta. Tube voltage was adjusted based on the patient’s body size; patients scanned at 100–120 kV were included. The gantry rotation time was 280 ms with a 16 cm axial coverage. The slice thickness and interval for reconstruction were 0.625 mm, and 40% adaptive statistical iterative reconstruction was standardized. The highest-quality images were selected from the available phases and used for reconstruction and final analysis using dedicated software.

### 2.6. Quantitative CCTA Analysis

Quantitative CCTA analysis was performed using the semiautomatic, validated software (Qangio CT Research Edition 3.2.0.13, Medis, Leiden, The Netherlands) [16]. All images were blinded to the observer, TRA. The American Heart Association 17-segment model was used as a guideline for plaque localization. The coronary artery tree was automatically extracted, and centerlines were placed using the software. Cross-sectional and longitudinal images of the lumen and vessel wall were automatically created, with careful manual adjustments when necessary. Segments with insufficient quality, with a lumen of less than 1.5 mm, or with low levels of contrast were excluded. Patients were excluded if they had less than two vessels of adequate quality or with severe artifacts that compromised image quality (*n* = 61) (Figure 1).

#### High-Risk Plaque Characteristics

**Figure 2:**
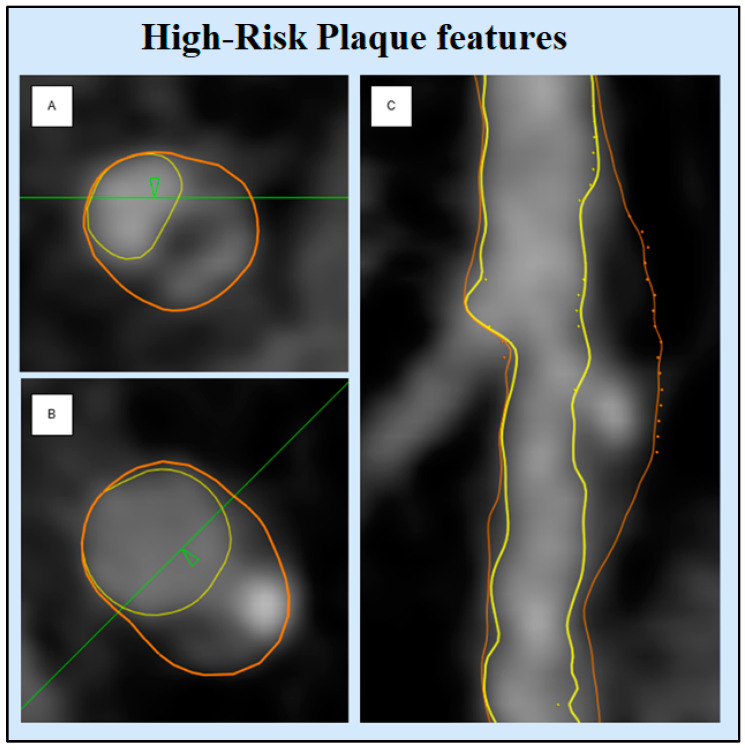
High-risk plaque features. This figure displays examples of high-risk plaque features: (**A**) Napkin-ring sign, (**B**) spotty calcification, (**C**) positive remodeling index. Images are derived from patient cases using QAngioCT Research Edition version 3.2.0.13 (Medis Medical Imaging) and include cross-sectional views for (**A**) and (**B**) and a longitudinal view for (**C**).

All vessels were examined for visible plaques and further evaluated using the semi-automatic software. Only lesions containing a minimum volume of LAP > 1 mm^3^ were evaluated [8]. The following high-risk plaque characteristics were extracted automatically per patient: total atheroma volume (TAV), vessel length, volumes of calcified plaque, non-calcified plaque, low-attenuation plaque, and lumen. Tissue volumes were extracted based on Hounsfield Unit (HU) values using a dynamic algorithm, converting HU thresholds into volumes according to luminal contrast densities [17]. The plaque burdens were calculated post-data extraction: percent plaque volume (PPV) per individual plaque component [(total calcified, non-calcified, and low attenuation plaque volume/total plaque volume) × 100%]. The coronary artery calcium score (CACS) was calculated using the Agatston method on non-contrast CCTA images [18]. HRP features, including NRS, RI, and SC, were evaluated for each LAP lesion identified (Figure 2). The napkin-ring sign was defined by a central area of low attenuation inside a semicircular area of higher attenuation (Figure 2A). Spotty calcifications were defined as small calcifications of <3 mm in any direction (Figure 2B). Positive remodeling index was calculated as the ratio of lesion plaque area to proximal reference area (Figure 2C) [19].

### 2.7. Statistical Analysis

All statistical analysis was performed using STATA IC 18 (StataCorp. 2023. College Station, TX, USA) and RStudio (RStudio Team, 2023 with R v. 4.3.2). Baseline characteristics are presented as means with standard deviations for continuous variables, and categorical variables are presented as totals and percentages of the total number per group. Patient and plaque level data were tested for normal distribution prior to analysis. Non-normally distributed variables are presented as medians and inter-quartile ranges. Student’s *t*-test was used to assess the difference in means of continuous variables between the two stratified groups (NGT and IGT). When appropriate, categorical parameters were described using the Chi-squared or Fisher’s test. The association between NGT and IGT groups with overall plaque burden and individual plaque components was tested using univariate and multivariate linear regression. HRP features were tested with univariate and multivariate logistic regression. All baseline characteristics with a *p*-value < 0.05 were tested using the Wald Test to determine whether they should be included in the final regression model.

## 3. Results

### 3.1. Clinical Characteristics

**Figure 3:**
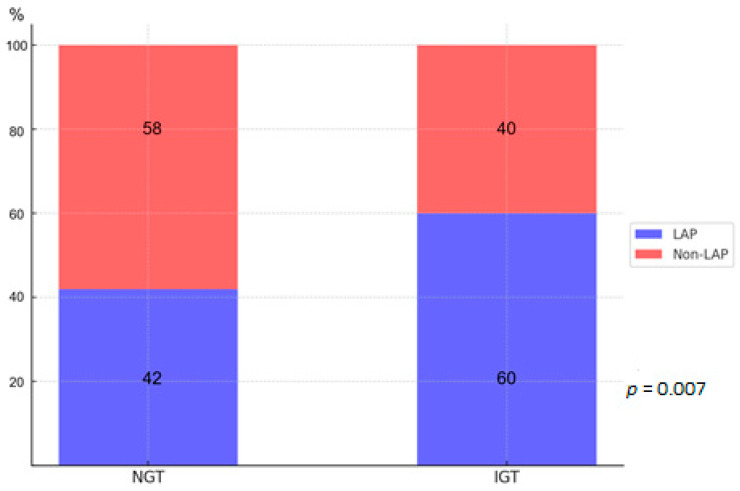
Shows the prevalence of low-attenuation plaque (LAP) lesions in the initial cohort of NGT and IGT patients (same abbreviations as in Figure 1).

The initial cohort included 312 patients, with 220 classified as NGT and 92 as IGT (Figure 1). LAP lesions were present in 42% of the NGT group and a significantly higher prevalence of 60% was found in the IGT group (*p* = 0.007) (Figure 3). Patients without LAP lesions were excluded (*n* = 164), leaving 148 patients with 202 lesions for further analysis (NGT = 93, IGT = 55). Table 1 shows the patient characteristics. Patients with NGT were significantly older than those with IGT (64.7 vs. 61 years, *p* = 0.02). Male sex, BMI, and smoking history were similar in the two groups. Lipid profiles were largely comparable, likely due to a greater use of statins in the IGT group compared with NGT, with the exception of HDL, which was higher in the NGT group. CRP and triglyceride levels were significantly elevated in the IGT group.

### 3.2. CCTA Findings

**Figure 4:**
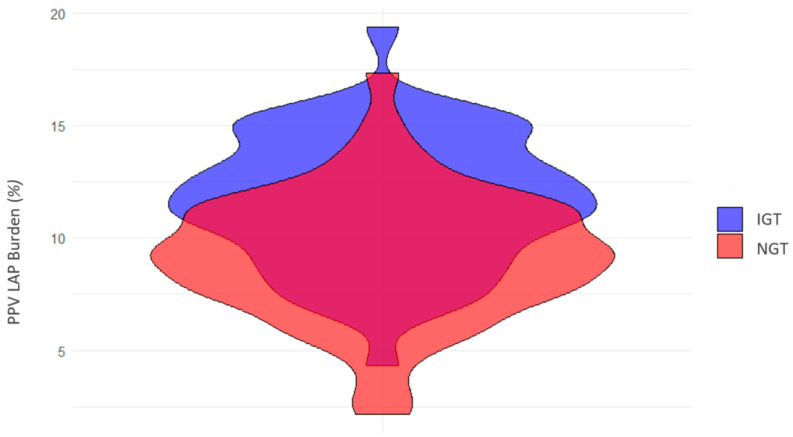
**Low-attenuation plaque burden according to glycemic tolerance.** This violin plot depicts the distribution of low-attenuation plaque burden (measured as a percentage of total plaque volume) in patients with impaired glucose tolerance (IGT, shown in blue) and normal glucose tolerance (NGT, shown in red). The width of each violin represents the density of patients with corresponding plaque burden values, highlighting the variation and overlap between the two groups.

Lesion length and TAV were similar in patients with NGT and IGT (Table 2). No differences were found in volumes and burdens of non-calcified and calcified plaques. A significantly higher volume and burden of LAP were found in patients with IGT compared with NGT (*p* = 0.01 and *p* = 0.02) (Figure 4). The HRP features RI and SC were numerically more prevalent in the IGT group but were not statistically significant. Patients with IGT exhibited a significantly higher frequency of NRS compared with NGT (12% vs. 5%, *p* = 0.02). The presence of two or more HRP features was more common in the IGT group but did not reach statistical significance.

### 3.3. Univariate and Multivariate Analysis

Table 3 presents a comparison of high-risk plaque characteristics. The univariate analysis demonstrated that the IGT group had significantly higher LAP volume and burden compared with the NGT group, this was also observed for the HRP feature NRS (all *p* = 0.02). In the multivariate model, the possible confounders (age, male sex, BMI, CRP, LDL, statin use, and smoking) were included. After adjusting for confounders, LAP burden remained significantly associated with IGT (β = 0.3, *p* = 0.03) and NRS remained significant in the multivariate analysis (odds ratio (OR) = 2.7, *p* = 0.02).

## 4. Discussion

This observational study aimed to compare the high-risk plaque characteristics in low-attenuation plaque lesions between patients with normal and impaired glucose tolerance. Our findings indicate that (1) the burden of low-attenuation plaque was significantly higher in IGT patients compared with NGT and (2) the napkin-ring sign was more frequent in IGT than NGT, while positive remodeling and spotty calcifications showed no significant differences. These results suggest that IGT may be associated with a more vulnerable plaque characteristic.

### 4.1. Prediabetes in a Clinical Context

Prediabetes is consistently recognized as a significant predictor of future CV events, with one study even suggesting it may pose a greater CV risk than well-managed T2DM [16,20]. Despite this elevated risk, no dedicated guidelines or therapeutic strategies currently target prediabetes for CV risk reduction. The 2023 ESC Guidelines [21] on the management of CV disease in patients with diabetes notably excluded prediabetes, focusing solely on manifest diabetes. This shift reflects an ongoing debate, as noted by Marx et al. [22], with some viewing prediabetes as a distinct clinical entity, while others attribute its risk to associated metabolic factors.

Our findings contribute to this debate, showing that among patients with LAP lesions, IGT was associated with a more vulnerable plaque characteristic compared with NGT. We found that patients with IGT exhibited a significantly higher burden of LAP, along with a greater prevalence of NRS. Importantly, these results persisted after adjusting for confounders, suggesting that IGT may play an independent role in influencing plaque vulnerability. However, the potential contribution of unmeasured risk factors cannot be excluded.

### 4.2. IGT vs. IFG: Different Paths to CVD?

The DECODE study [23] further highlighted the stronger CV risk associated with IGT compared with IFG. This distinction may be reflected in the differences in pathophysiology. Elevated FPG primarily indicates hepatic dysfunction in glucose regulation. In contrast, elevated 2-hPG reflects postprandial glucose dysregulation caused by decreased pancreatic insulin secretion and increased insulin resistance in peripheral tissues [22]. These mechanistic differences likely explain the stronger association of IGT with CV risk and underscore the importance of OGTT in identifying high-risk populations.

### 4.3. Plaque Burden in Prediabetes

To the best of our knowledge, this study is the first to demonstrate an association between IGT and adverse plaque characteristics using CCTA measurements. We found that IGT was associated with a higher burden of LAP. Supporting our findings, Gurudevan et al. [24] reported that both IFG and T2DM were associated with higher plaque burdens compared with NGT, and they found no significant differences between IFG and T2DM. However, unlike Gurudevan et al., our study focused specifically on IGT, which has been shown to have a stronger association with cardiovascular disease risk [25]. Similarly, Kuhirara et al. [26] used angioscopy in patients with known CAD and found that plaque vulnerability was more advanced in patients with prediabetes compared with patients with NGT. They also observed similar findings among those with prediabetes and T2DM. However, direct comparisons are limited by the difference in the definition of prediabetes, as Kuhirara et al. used FPG and HbA1c levels. Furthermore, they assessed plaque vulnerability by angioscopy.

Sassa et al. [27] observed similar levels of soft plaque characteristics and remodeling indexes in patients with IGT and T2DM, but they did not include an NGT group for comparison, which limits the direct comparability to our study. A previous study from our group [28] demonstrated an association between insulin resistance, measured by HOMA-IR, and necrotic plaque volume. However, we did not find a significant link between prediabetes, defined by OGTT, and necrotic core volume. Notably, we did not differentiate between IFG and IGT. In contrast to our current findings, the Paradigm Study [29] and Prospect Study [30] reported limited associations between prediabetes and plaque burden or progression, particularly over short follow-up periods. These findings may reflect the need for longer-term studies to capture the cumulative effects of prediabetes on plaque development. Additionally, the use of imaging modalities such as intravascular ultrasound in the Prospect Study may limit comparability with our findings.

### 4.4. High-Risk Plaque Features in Prediabetes

Quantitative plaque characteristics, such as plaque burden, are associated with the qualitative assessment of HRP features [31]. In our study, the NRS was more frequent in patients with IGT compared with NGT, while no significant differences were observed for positive remodeling and spotty calcifications. These findings are consistent with a study [32] that used OCT in patients undergoing percutaneous coronary intervention. The study showed that patients with IGT exhibited more vulnerable non-culprit lesions, characterized by a larger lipid core and a thinner fibrous cap, compared with patients with NGT. These characteristics were similar to those observed in patients with T2DM. However, that study consisted of a high-risk population undergoing intervention, whereas our study included low-risk patients with suspected stable CAD.

Interestingly, the Miami Heart Study [33], which included a large population of over 2300 asymptomatic adults without known CAD, found that worsening glycemic status, as measured by HbA1c, was associated with a higher prevalence of HRP features. Specifically, patients with prediabetes had an odds ratio of 1.7 for HRP features, while those with T2DM had an odds ratio of 2.5, compared with NGT. Our study population lies somewhere between these two cohorts, bridging the gap between high-risk intervention patients and low-risk asymptomatic individuals.

### 4.5. Methodological Considerations

A limitation lies in the cross-sectional nature of the study, which limits the ability to establish causal relationships between glycemic tolerance and plaque characteristics. Additionally, there was a delay between CCTA and blood sampling, which could potentially introduce variability. The OGTT was based on a single measurement, and while OGTT is widely used, the reproducibility for the 2h plasma glucose value has been questioned with the report of an intrapersonal coefficient of variation of 16.7% [34]. This variability could influence the classification of glycemic tolerance in some individuals. Plaque measurements were performed using validated semi-automatic methods with manual corrections when needed, which might introduce errors. Finally, the sample size and selection criteria, focusing on a specific subset of patients (only those with LAP lesions), may limit the generalizability of the findings to broader populations.

## 5. Conclusions

In patients with suspected stable coronary artery disease who exhibited coronary low-attenuation plaque lesions, those with impaired glucose tolerance had a higher low-attenuation burden and a greater frequency of napkin-ring signs compared with patients with normal glucose tolerance. These findings may provide insight into why prediabetes is frequently observed in patients with acute myocardial infarction. However, further research is needed to explore the underlying mechanisms and potential clinical implications of these associations, particularly regarding their role in predicting cardiovascular events.

## Figures and Tables

**Table 1:** Patient characteristics according to glycemic tolerance.

	NGT *n* = 93	IGT *n* = 55	*p*-Value
Age, years, *n* (SD)	64.7 (8.8)	61.0 (9.4)	0.02
Sex, male, *n* (%)	62 (67%)	42 (76%)	0.2
BMI, kg/m^2^ (SD)	27.4 (6.0)	28.9 (4.3)	0.1
Systolic BP, mmHg (SD)	144 (25)	150 (22)	0.2
Diastolic BP, mmHg (SD)	80 (13)	83 (12)	0.2
Never smoker, *n* (%)	44 (47%)	20 (36%)	0.7
Former smoker, *n* (%)	38 (41%)	25 (45%)	0.1
Active smoker, *n* (%)	11 (12%)	9 (16%)	0.4
Family history of CVD, *n* (%)	29 (31%)	22 (40%)	0.3
Fasting glucose, mmol/L (SD)	5.6 (0.3)	6.1 (0.5)	<0.001
120 min glucose, mmol/L (SD)	5.7 (1.1)	8.7 (1.2)	<0.001
HbA1c, mmol/mol (SD)	35 (4)	37 (3)	0.03
Pre-diabetic HbA1c, *n* (%)	34 (37%)	18 (33%)	0.7
Total cholesterol, mmol/L (SD)	5.0 (1.1)	4.6 (1.2)	0.06
HDL, mmol/L (SD)	1.5 (0.4)	1.3 (0.3)	<0.001
LDL, mmol/L (SD)	2.9 (1.0)	2.6 (1.1)	0.2
Triglycerides, mmol/L (SD)	1.3 (0.8)	1.8 (0.9)	0.003
CRP, mg/L (SD)	2.1 (1.9)	3.1 (2.8)	0.02
Antihypertensive medication, *n* (%)	49 (53%)	32 (60%)	0.2
Statins, *n* (%)	26 (28%)	26 (47%)	0.02
Medical history			
AMI	2 (2%)	2(4%)	0.6
PCI or CABG	1 (1%)	1 (2%)	0.7
Stroke	3 (3%)	2(4%)	0.8
Heart failure	1(1%)	0	0.6
Total CACS, (IQR)	73 (4–233)	76 (1–214)	0.5
Inflammatory disease	6(6%)	4(7%)	0.8
**LAP lesions:**			
NCP, *n* (%)	36 (30%)	30 (38%)	0.2
Mixed mostly fibrous, *n* (%)	72 (59%)	42 (53%)	0.4
Mixed mostly calcified, *n* (%)	12 (10%)	8 (10%)	0.4

Data are presented as mean ± standard deviation (SD), median + interquartile range (IQR), or counts (*n*) + proportions (%). BMI = body mass index, BP = blood pressure, CVD = cardiovascular Disease, HbA1c = glycated Hemoglobin A1c, HDL = high-Density Lipoprotein, LDL = low-density lipoprotein, CRP = C-Reactive Protein, CACS = coronary artery calcium score, LAP = low-attenuation plaque, NCP = non-calcified plaque.

**Table 2:** High-risk plaque characteristics according to glycemic tolerance.

	NGT *n* = 122	IGT *n* = 80	*p*-Value
*Plaque location*			
Left anterior descending artery	70 (57%)	42 (53%)	**-**
Circumflex artery	18 (15%)	5 (6%)	**-**
Right coronary artery	34 (28%)	33 (41%)	**-**
**Plaque metrics**			
Lesion length, mm	17.5 (5.4)	17.4 (5.8)	0.9
TAV, mm^3^	155.6 (72.9)	157.5 (76.0)	0.9
Degree stenosis %	31%(18, 59)	37(16, 62%)	0.3
**Plaque volumes, mm^3^**			
Calcified	25.7 (37.7)	22.1 (27.5)	0.5
Non-calcified	109.8 (48.2)	114.3 (61.4)	0.6
Low-attenuation	12.6 (9.7)	16.5 (12.5)	0.01
**Plaque burdens, %**			
Calcified	14.2 (14.2)	12.8 (11.8)	0.5
Non-calcified	72.3 (12.8)	73.1 (10.5)	0.7
Low-attenuation	8.623 (5.9)	10.8 (6.8)	0.02
**HRP features, *n* (%)**			
Positive remodeling	21 (17%)	15 (19%)	0.8
Spotty calcification	28 (23%)	26 (32%)	0.1
Napkin-ring sign	5 (5%)	10 (12%)	**0.02**
HRP ≥ 2	46 (38%)	39 (49%)	0.1
HRP ≥ 3	8 (7%)	10 (13%)	0.1

Data are presented as means ± SD or total count and percentage. HRP ≥ 2/3 refers to lesions where two or three high-risk plaque features are present. TAV = total atheroma volume, HRP = high-risk plaque.

**Table 3:** Linear and logistic regression models for high-risk plaque characteristics according to glycemic tolerance.

	NGT vs. IGT	*p*-Value	NGT vs. IGT	*p*-Value
	Univariate		Multivariate	
	β		β	
**Plaque volumes, mm^3^**				
Calcified	0.3	0.08	0.02	0.1
Non-calcified	4.4	0.5	0.04	0.6
Low-attenuation	3.7	0.02	2.9	0.06
**Plaque burdens, %**				
Calcified	−0.1	0.7	0.3	0.3
Non-calcified	0.8	0.7	0.3	0.9
Low-attenuation	0.4	0.02	0.3	0.03
**HRP features, *n***	**OR**		**OR**	
Positive remodeling	1.1	0.8	1.04	0.9
Spotty calcifications	1.6	0.1	1.4	0.4
Napkin-ring sign	2.7	0.02	2.2	0.04

The multivariate analysis was adjusted for age, male sex, BMI, CRP, LDL, statin use, and smoking. HRP = high-risk plaque.

## Data Availability

The data presented in this study are available on request from the corresponding author.

## Supplementary Materials

The following supporting information can be downloaded at: https://www.mdpi.com/article/10.3390/jcdd12020037/s1, Table S1. Patient characteristics according to glycemic tolerance; Table S2. Plaque burden and High-risk plaque characteristics according to glycemic tolerance.
