# Supplementary material for: High-Risk Plaque Characteristics in Patients with Suspected Stable Coronary Artery Disease and Impaired Glucose Tolerance: A Coronary Computed Tomography Angiography Study
Source: J Cardiovasc Dev Dis. 2025 Jan 22;12(2):37. doi: 10.3390/jcdd12020037 (PMC11856177; doi:10.3390/jcdd12020037)
Supplement: Supplementary file 1 [file jcdd-12-00037-s001.zip › jcdd-3390036-supplementary.pdf]

Table S1. Patient characteristics according to glycemic tolerance.

	NGT<br><br>*N = 93*	*IGT*<br><br>*N = 55*	*T2DM*<br><br>*N=15*	*p-value*
Age, years	64.7(8.8)	61.0(9.4)	60.1(9.1)	0.06
Sex, male	62(67%)	42(76%)	12(80%)	**0.006**
BMI	27.4(6.0)	28.9(4.3)	28.6(4.2)	0.2
Systolic BP mmHg	144(25)	150(22)	145(21)	0.3
Diastolic BP mmHg	80(13)	83(12)	80 (12)	0.2
Never smoker	44(47%)	20(36%)	5(33%)	0.3
Former smoker	38(41%)	25(45%)	7(47%)	0.8
Active smoker	11(12%)	9(16%)	3(20%)	0.8
Fam. History of CVD	29(31%)	22(40%)	6(40%)	0.4
Fasting glucose	5.6(0.3)	6.1(0.5)	-	**<0.001**
120min glucose	5.7(1.1)	8.7(1.2)	-	**<0.001**
HbA1c	35(4)	37(3)	52 (9)	**<0.001**
Total cholesterol	5.0(1.1)	4.6(1.2)	3.5(0.7)	**0.002**
HDL	1.5(0.4)	1.3(0.3)	1.2(0.3)	**0.001**
LDL	2.9(1.0)	2.6(1.1)	1.8(0.5)	**0.007**
Triglycerides	1.3(0.8)	1.8(0.9)	1.6(1.0)	**0.005**
CRP	2.1(1.9)	3.1(2.8)	2.1(2.6)	0.2
Antihypertensive medication	49(53%)	32(60%)	11(73%)	0.2
Statins	26(28%)	26(47%)	13(/87%)	**0.006**
Total calcium score	73(4-233)	76(1-214)	243(74-568)	0.7
Lesions				
NCP	36 (30%)	30 (38%)	6(27%)	0.4
Mixed mostly fibrous	72 (59%)	42 (53%)	13(59%)	0.6
Mixed mostly calcified	12 (10%)	8 (10%)	3(14%)	0.8

Table S2 Plaque burden and High-risk plaque characteristics according to glycemic tolerance.

	NGT N = 122	*IGT* N = 80	*T2DM* N=22	*p-value*
Lesion length, mm	17.5 (5.4)	17.4 (5.8)	17.5(7.1)	0.9
TAV	155.6 (72.9)	157.5 (76.0)	150.1(67.9)	0.9
Calcified plaque vol	25.7 (37.7)	22.1 (27.5)	28.(36.3)	0.5
Non-calcified plaque vol	109.8 (48.2)	114.3 (61.4)	106.2(48.2)	0.6
Low-attenuating plaque vol	12.6 (9.7)	16.5 (12.5)	13.6(14.7)	0.1
PPV calcified	14.2 (14.2)	12.8 (11.8)	16.1(17.4)	0.5
PPV non-calcified	72.3 (12.8)	73.1 (10.5)	72.7(12.9)	0.7
PPV Low-attenuating	8.623 (5.9)	10.8 (6.8)	9.6(9.1)	0.2
Positive remodeling	21(17%)	15(19%)	3(14%)	0.8
Spotty calcifications	28(23%)	26(32%)	5(22%)	0.1
Napkin-ring sign	5(5%)	10(12%)	2 (9%)	**0.05**

Data are presented as means ±SD or total count and percentage. HRP ≥ 2/3 refers to lesions where two or three high-risk plaque features are present. TAV= Total atheroma volume, HRP= High-risk plaque.
